# Characterization of Collagen from Three Genetic Lines (Gray, Red and F1) of *Oreochromis niloticus* (Tilapia) Skin in Young and Old Adults

**DOI:** 10.3390/molecules27031123

**Published:** 2022-02-08

**Authors:** Nataly Reátegui-Pinedo, David Salirrosas, Linda Sánchez-Tuesta, Claudio Quiñones, Segundo R. Jáuregui-Rosas, Gabriela Barraza, Angelita Cabrera, Carmen Ayala-Jara, Renata Miliani Martinez, André Rolim Baby, Zulita Adriana Prieto

**Affiliations:** 1 Department of Biological Sciences, Faculty of Biological Sciences, Universidad Nacional de Trujillo, Juan Pablo II Av., Trujillo 13008, Peru; pinedo29@outlook.com (N.R.-P.); rsalirrosas@unitru.edu.pe (D.S.); lsanchezt@unitru.edu.pe (L.S.-T.); acabrera@unitru.edu.pe (A.C.); 2 School of human Medicines, Faculty of Human Medicine, Universidad Privada Antenor Orrego, Av. América Sur 3145, Trujillo 13008, Peru; claudioqc24@gmail.com; 3 Department of Physics, Faculty of Physical Sciences and Mathematics, Universidad Nacional de Trujillo, Juan Pablo II Av., Trujillo 13008, Peru; sjauregui@unitru.edu.pe; 4 Department of Agricultural Sciences, Faculty of Agricultural Sciences, Universidad Nacional de Trujillo, Juan Pablo II Av., Trujillo 13008, Peru; gbarraza@unitru.edu.pe; 5 Department of Pharmacotechnics, Faculty of Pharmacy and Biochemistry, Universidad Nacional de Trujillo, Juan Pablo II Av., Trujillo 13008, Peru; cayala@unitru.edu.pe; 6 Department of Pharmacy, Faculty of Pharmaceutical Sciences, University of São Paulo, São Paulo 05508-000, Brazil; renata.martinez@usp.br

**Keywords:** tilapia, collagen, fish skin, *Oreochromis niloticus*

## Abstract

From tilapia (*Oreochromis niloticus*) farming, the by-products have been identified as a source of collagen that could be used for the development of dermocosmetics or pharmaceutical products. However, the characteristics of collagen related to a specific strain or culture must be well defined prior to its application. Collagen was extracted from the skin of three strains of tilapia: red YY males (YY: two Y-type sex chromosomes), XX gray females, and the F1: offspring of crossing red YY males with XX gray females; at different ages in the adult phase, using acetic acid and pepsin enzyme. The characteristics of acid-soluble collagen (ASC) and pepsin-soluble collagen (PSC) were shown by SDS-PAGE band profiles to be similar to bovine collagen type I (SIGMA), the PSC of gray tilapia being more fragile to temperature changes, consistent with the results of fractional viscosity. The characteristics of the F1 progeny were prioritized for being a commercially productive and sustainable source for the extraction of collagen, and the ASC form, being the one with the greatest stability and advantage over PSC, of importance to our investigations, leads to a controlled digestion as in the case of peptide induction, and also in the development of natural products in the pharmaceutical and/or dermocosmetic industry. Evaluations of the triple helix structure by FT-IR, X-ray diffraction and UV–visible spectroscopy give similar results between the strains: red, gray, and F1, and between ages in the adult form F1 (15, 24, and 36 months of age). Consequently, the skin of tilapia in adult form is recommended sustainably for up to 24 months of age where the collagen is obtained with the use of acetic acid without enzymatic treatment.

## 1. Introduction

Pharmaceutical, cosmetic, and food industries’ increasing collagen demands has brought about the need for a methodologic strategy implementation to increase the amount and quality of collagen available to be used as an ingredient. The main sources of collagen are the skin and other residues of pigs, cattle, and birds [1]. However, due to the probability of risk of disease transmission to humans, it is important to consider other sources, such as fish and mollusks [2].

To obtain collagen from fishes in a sustainable way, it is important to prioritize and consider fish farming. This activity allows the breeding handling to be controlled and also contaminant-free raw material to be obtained, according to biosecurity international standards. Catching fish, either on the continent or from the sea, does not guarantee total health. There are reports about contaminants being present in different oceans, lakes, and rivers around the world. Some are heavy metals, whose concentrations can exceed the allowable values, such as in East China marine sediment [3]. In other cases, the pollutants are microplastics, such as those detected in the sediment of the Baltic Sea [4], in the Atlantic Ocean [5], and on the Japanese shore [6]. In this sense, aquaculture is a promising alternative to guarantee that high-quality ingredients are obtained from good management practices applied in aquatic resources.

From farming species, *Oreochromis niloticus* “tilapia” stands out, since it is an easy to breed freshwater specie. It has demonstrated adaptability to climatic changes due to its spread in tropical and subtropical environments around the world [7]. It also ensures sustainability over time since its meat and skin are sources of collagen type I and other bioactive compounds [8]. This collagen is distinguished by its structural integrity stability, correlated with proline and hydroxyproline content [9,10,11,12].

Content change in these amino acids is due to environmental conditions, either nutritional or disease, as well as individuals’ age, lines, or genetic species. From research on seven marine fish species that live in warm and cold temperatures, it was confirmed that there was a positive association among amino acid (proline and hydroxyproline) content and thermal stability, and negative correlation with serine content variation [13]. Regarding age, Thompson and Contin [14] observed changes in hydroxyproline concentration in *Thalassoma bifasciatum* collagen—higher in young ones, and, after six weeks, a gradual reduction in hydroxyproline concentration correlated with either the amount of collagen or glycation process. However, in *Lates niloticus*, collagen change was not associated between young and old fish [15].

Among the indicators of the age of the fish, the maturity of the gonads is considered the most reliable. In tilapia males, the reproductively active fish at the age of 9 months (young adults) present their gonads of uniform thickness and pink coloration, not so in the old adults. From 2 years or more, gonadal segmentations appear, which are accentuated as age advances and that are correlated with a decrease in sperm quality [16]. The authors in [17] reported a decrease in the number of offspring after two and a half years. These characteristics would be typical of old adult fish. The structural changes in collagen in tilapia as a function of age has not yet been studied.

By way of exposure, we focused on determining red YY, grey, and F1 progeny collagen characteristics of *O. niloticus* crosses, as well as on variations in collagen characteristics in fish of different ages. We obtained ASC and PSC collagen by treatments with acetic acid and pepsin enzyme, and characterization was performed by denaturation temperature (viscosity), hydroxyproline quantification, ultraviolet (UV) scanning, Fourier transform infrared spectroscopy (FTIR), and X-ray diffraction.

## 2. Results

**Figure 1:**
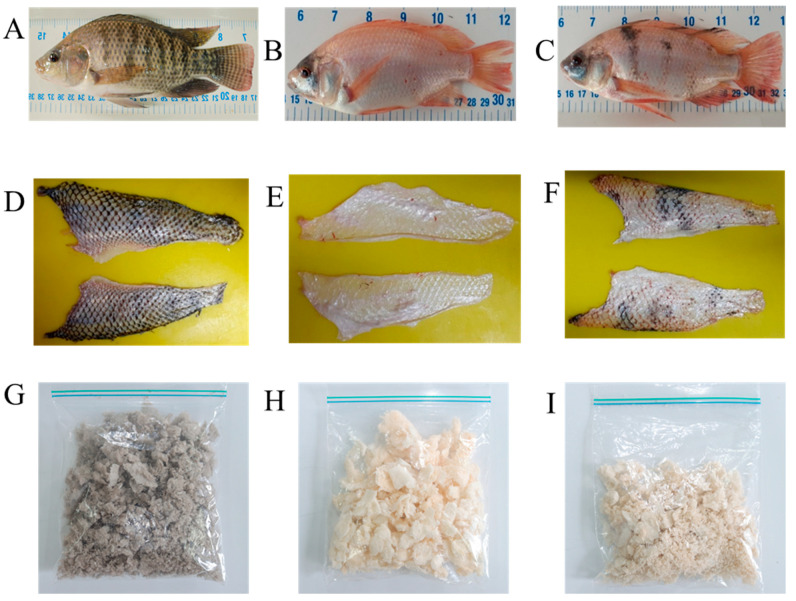
Tilapia genetic strain (*Oreochromis niloticus*). (**A**) Gray XX, (**B**) Red YY, (**C**) F1 XY, and (**D**), (**E**), and (**F**) are gray, red, and F1 skins, respectively; (**G**), (**H**), and (**I**) are lyophilized collagens from gray, red, and F1 fish, respectively.

In Figure 1, we can observe gray tilapia, red tilapia, and generation F1 (known as “hybrid”). Lead-colored freeze-dried collagen corresponds to gray tilapia, light pink to the red tilapia, and the pink color of a shade lighter than red tilapia is from the F1 fish. The percentage of skin obtained from fish at different ages was in the range of 3.01 to 3.55% of body weight (Table 1). Collagen output obtained by ASC and PSC methods, and among the lineages, presented no differences (~20%) (Table 2).

**Figure 2:**
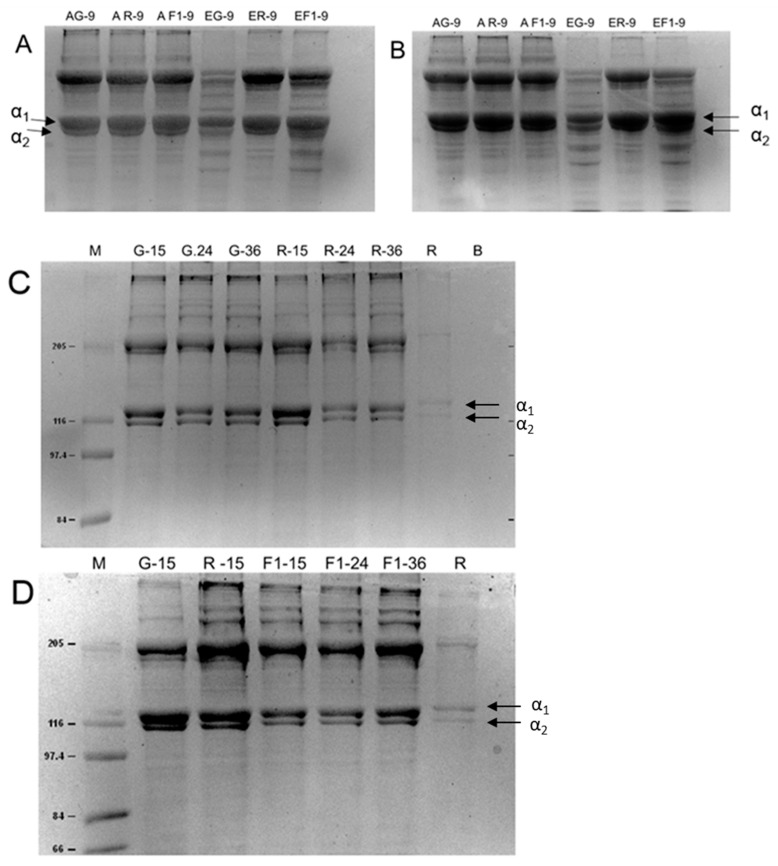
SDS-PAGE electrophoresis of collagen samples from red, gray, and F1 tilapia (*Oreochromis niloticus*) skin from the cross of red YY males with XX gray females. (**A**). Without mercaptoethanol from 9-month-old fish collagen ASC samples, (AG-9: ASC-Gray, AR-9: RED ASC, AF1: ASC-F1) and by the PSC collagen (EG-9: PSC-Gray, ER-9: PSC RED, EF1: PSC-F1); (**B**). With mercaptoethanol from 9-month-old fish collagen samples; (**C**). Collagen ASC, M: molecular weight marker, G-15: gray 15 months, G-24: gray 24 months, G-36: gray 36 months, R-15: Red 15 months, R-24: Red 24 months, R-36: red 36 months R: bovine skin collagen, B: Run buffer (control). (**D**). Collagen ASC, M: molecular weight marker, G-15: gray 15 months, R-15: Red 15 months, F1-15: F1 15 months, F1-24: 24 months, F1-36: F1 36 months. R: bovine skin collagen. α1 and α2 chains.

The lyophilized samples the ASC and PSC collagen were analyzed by SDS-PAGE electrophoresis. The band profile of the samples was similar to calf skin type I collagen, used as reference. The bands that typified the α1 and α2 chains were evident. The intensity of α1 chain band was twice that of the α2 chain, and the molecular weights were between 117 and 120 kDa for the α1; in the range of 110 to 112 kDa for the α2; and ~200 kDa for the β chain (Figure 2). The profile of bands between the gray, red, and F1 tilapias the ASC were similar to each other. However, in the collagen PSC, additional bands were observed in the gray tilapia collagen, to a greater degree, followed by F1 collagen, but not in the red tilapia. In all cases, the treatment with mercaptoethanol during the electrophoretic run did not generate changes in the band profile in any of the collagen samples. The collagen band profile obtained by the acid acetic method showed similarity among the red, gray, and F1 lineages and amongst the different ages.

**Figure 3:**
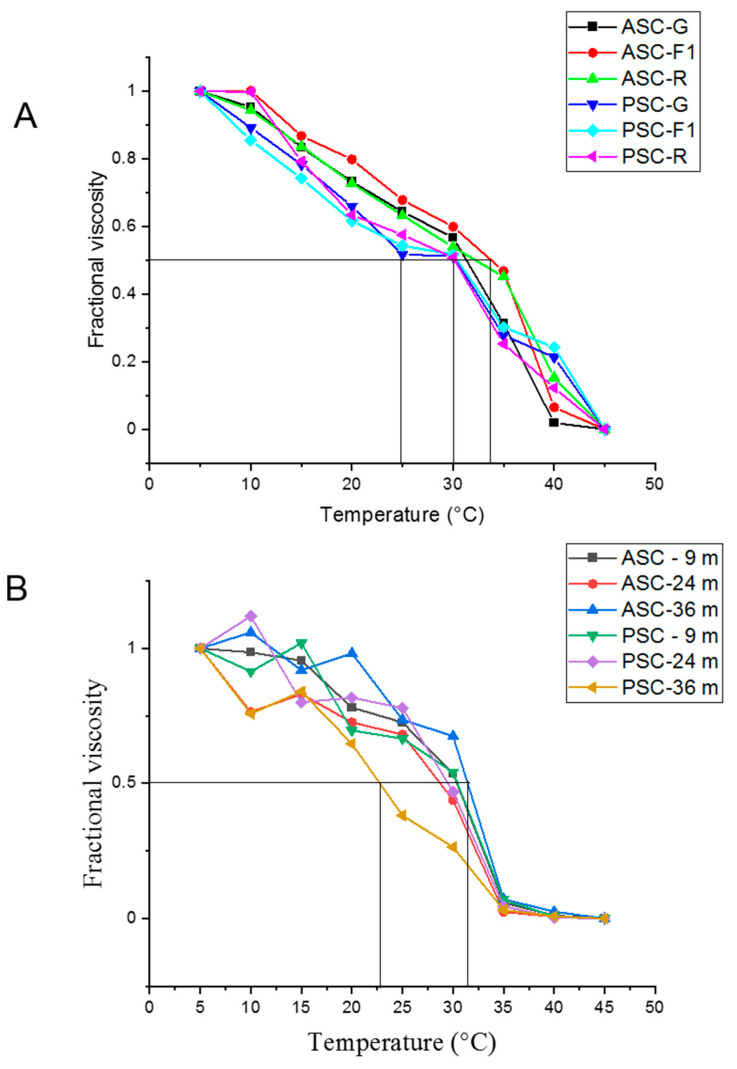
Thermal denaturation curves of tilapia skin collagen. (**A**). Collagen ASC and PSC of red, gray, and F1 strains (9-months old); (**B**). ASC and PSC collagens from F1 progeny ages 9, 24, and 36 months.

The degree of collagen stability was determined considering the denaturation temperature (τd), when the changes in the fractional viscosity reached the value of 0.5 [18]. The collagen denaturation temperatures of 9-month-old fish obtained by acetic acid and pepsin treatments were similar, at ASC ~32, ~33, and ~34 °C in the gray, red, and F1, respectively; the τd of the collagens PSC in the three lines presented the same value (~30 °C) (Figure 3A). Comparison of fractional viscosity values of collagen from F1 fish of 9, 24, and 36 months of age showed denaturation values at a temperature of approximately 32 °C, except for collagen PSC from 36-month-old fish, where the denaturation temperature was ~23 °C (Figure 3B).

**Figure 4:**
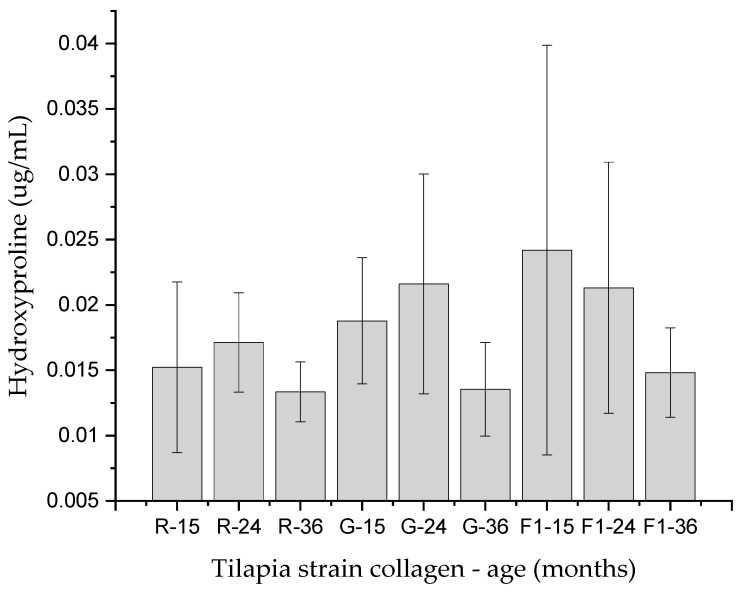
Hydroxyproline (µg/mL) in the skin collagen ASC of tilapia strains of 15, 24, and 36 months from adult individuals. No significant differences.

Figure 4 shows the concentration of hydroxyproline (µg/mL) in the skin collagen ASC of tilapia strains of 15, 24, and 36 months from adult individuals without significant differences (*p* > 0.05).

**Figure 5:**
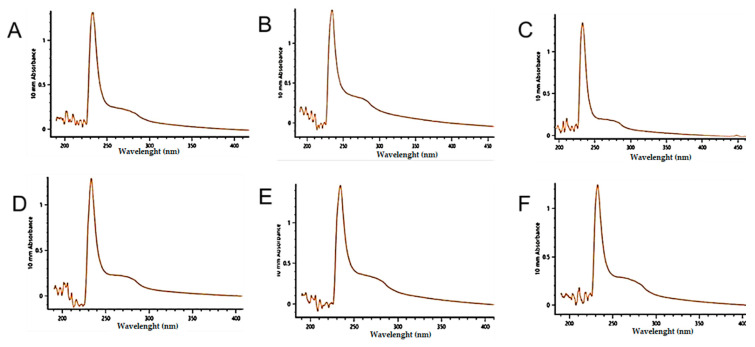

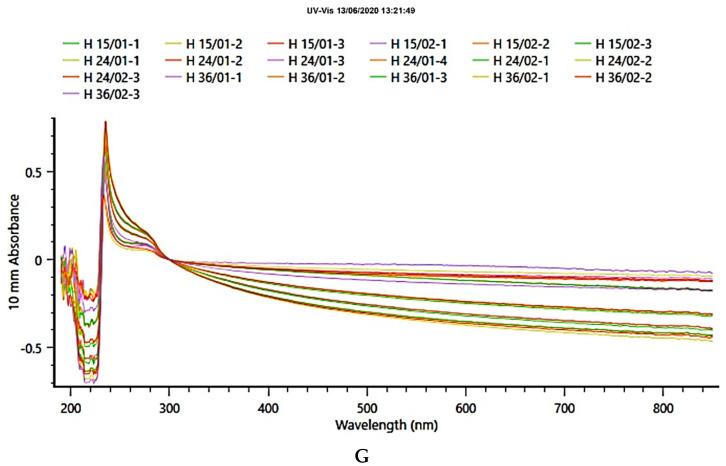
UV absorption spectrum of *Oreochromis niloticus* “tilapia” skin collagen samples (9 months old). (**A**). Red ASC (233 nm); (**B**). Gray ASC (234 nm); (**C**). F1 ASC (233 nm); (**D**). Red PSC (233 nm); (**E**). Gray PSC (234 nm); (**F**). F1 PSC (232 nm). (**G**): UV-visible spectrum of collagen from F1 (H) aged: 15, 24 and 36 months old.

The UV absorption profile of the lyophilized collagen samples from the three lineages (9 months old) had the highest peaks between 232 and 234 nm (Figure 5A–F). The curves of the set of collagen spectra at ages 15, 24, and 36 months of age of the F1 generation presented the same distribution (Figure 5G).

**Figure 6:**
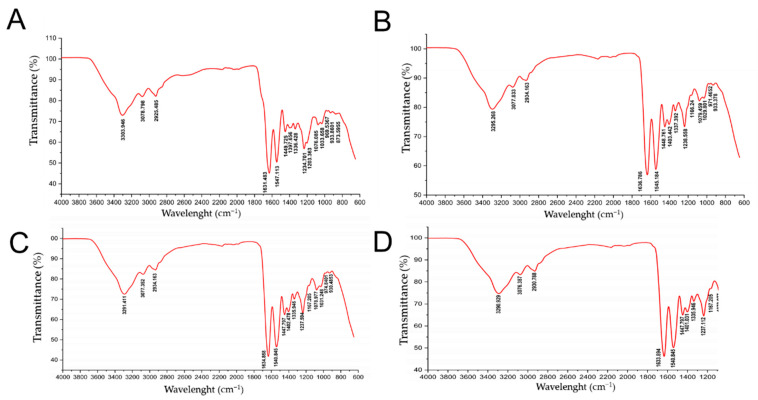
FTIR profile of collagens extracted in 0.5 M acetic acid from tilapia skin. (**A**). Collagen from calf skin (SIGMA); (**B**). Collagen from F1 progeny; (**C**). Red strain collagen; (**D**). Gray strain collagen.

The collagens ASC and PSC infrared spectrums were found at 3290 to 3295 cm^−1^ in the three lineages (red, gray, and the F1 progeny). The FTIR spectra distribution of tilapia skin collagen showed amide A band at 3290 cm^−1^ in the three strains at 9, 24, and 36 months of age, and similarly for the band amide B, amide II, and amide III, at 2940, 1640, and 1240 cm^−1^, respectively. Values in bovine skin collagen (reference sample) were recorded for amides A, B, I, II, and III at 3304, 2925, 1631, 1547, and 1235 cm^−1^, respectively (Figure 6 and Table 3).

**Figure 7:**
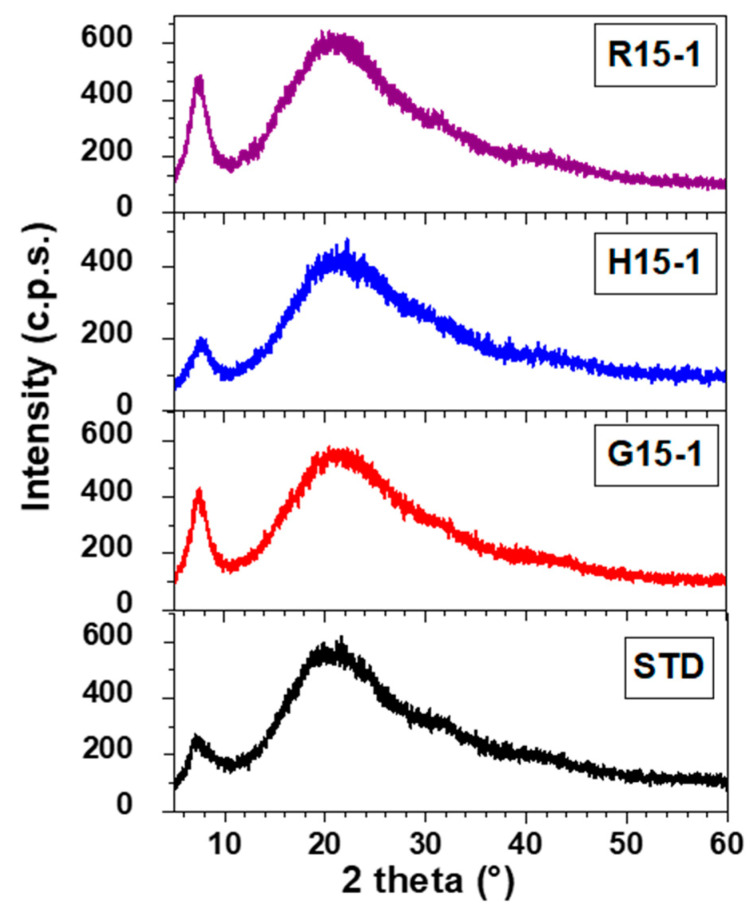
X-ray diffraction diagram of skin collagen from *Oreochromis niloticus*. R15-1: red tilapia 15-months old, H15-1: F1 (XY from red YY x gray XX) 15-months old, G15-1: gray tilapia 15-months old and STD: collagen skin calf type I (SIGMA).

The X-ray diffraction showed two peaks for the three tilapia strains, with slight variations. For the red one, they were 7.47° and 20.96°; for the gray, 7.47° and 21.17°; and for the F1 (hybrid), 7.78° and 21.28°. Values were close to type I bovine collagen (Figure 7). The X-ray diffraction diagrams of tilapia skin collagen samples at 9, 15, 24, and 36 months present similar values, without differences due to age, two characteristic collagen peaks were recorded for all samples (Table 4).

## 3. Discussion

The tilapia lineages identified mainly by the color of the skin (gray and red) are the most cultivated ones in different parts of the world [19] and whose phenotypic and genetic differentiation has been demonstrated [20,21]. Phenotypically, the variation in skin color is due to the existence and arrangement of chromatophores typical of teleosts [22], which, in turn, would be conditioned by their genetic and nutritional factors, temperature, and luminosity [23]. The quality characteristics of the collagen may vary depending on the skin pigmentation, in addition to other factors, such as the age of the fish.

The yield of the skin weight (between 3.01 and 3.55% of the total weight) was similar in function at the ages of 9, 15, and 36 months for the three genetic lines. Investigations on the proximal analysis of tilapia skin revealed the presence of proteins in 72.47% of the whole skin, and in the collagen extracted from the skin, the percentage of proteins was reported in 97.18% [24]. This value constitutes an indicator of the elimination of lipids during the collagen extraction process. Research has been carried out on the content of proteins and lipids in the skin of fish species that are the main sources for the extraction of collagen, due to the high protein content and the low amount of lipids, as registered for tilapia, at 0.79% by Le et al. [25] and 3.27% by Li et al. [18]. Regarding the yield of the collagen extracted in the present investigation, no differences were found among the lines (gray, red, and the progeny F1). The registered values were similar to those reported by Li et al. [18]. According to Le et al. [25], the collagen yield of tilapia skin was 12.50%, a lower percentage than that registered.

Considering that commercial production is sustainable with the F1 generation due to the characteristics of more homogeneous growth than the red and gray lines and, consequently, having a higher yield. On the other hand, ASC collagen has greater thermal stability compared to PSC. These are the reasons why it has been preferred to carry out the evaluations at different ages in the F1 progeny.

The band profiles obtained by SDS-PAGE electrophoresis of the red and gray tilapia skin collagens and the F1 progeny were like those recorded in the bovine type I collagen sample. The α chain was present in twofold greater intensity than the α2 chain, which is typical for type I collagen, and it was observed for all collagens from tilapia skins (gray, red, and F1 strains), as well as in all fish of different ages. Other bands of higher molecular wight were outlined corresponding to the β chains with ~200 kDa and the γ chains of higher molecular weight [18,26]. The profile of bands with and without β-mercaptoethanol in the SDS-PAGE did not generate differences among the samples due to the existence of a low amount of disulfide bridges between the α and β chains [27]. However, the effects of the treatment with pepsin in the collagen extraction process generated greater degradation in the gray tilapia collagen, evidenced by the presence of bands of lower molecular weight, with greater intensity than in the collagen ASC of the three lines obtained.

Separation of α subunits by ultracentrifugation of tilapia skin gelatin reported a1 and a2 units in relative amounts of 50.6 and 23.3%, respectively [28]. Kimura et al. [29] reported the presence of a third chain (α3) in tilapia in lower proportions than in other teleosts detected only by CM-cellulose chromatography. The migration of the α3 chain is very close to that of the α1 chain, detected in zebrafish collagen; the presence of three bands evident by electrophoresis and identified by mass spectroscopy correspond to the heterotrimers formed with the presence of three α1, α2 units, and α3 [30]. The presence of the α3 chain, in variable amounts between species and absent in others, is an indicator of phylogenetic variants. So too, the presence of the α3 chain in a low proportion in tilapia, could be an advantage in compatibility with human collagen.

In collagen extraction with pepsin treatment, gray tilapia skin collagen was more degraded than red tilapia and F1 collagen, evidenced by the presence of lower molecular weight bands. This degradation does not occur with the collagen ASC extracted, which, by keeping its structure intact, has an advantage in the use of biomedical materials. 

The thermal stability of the collagen of the three genetic lines at 9 months of age was similar in both ASC and PSC collagens; in this sense, referring to the absence of effects by the extraction methods, they were consistent with the results of Sun et al. [31]; however, the values obtained in our investigation were lower by ~2 °C; this variation may be due to the rearing conditions, as the fish were kept in subtropical conditions (23 + 3 °C). According to the reports of Akita et al. [13], the τd is influenced by the temperature of the water; when the fish inhabit warm waters, the τd is higher than in those fish that inhabit cold waters: in warm water species in the marine environment of 22.9 + 4.9 °C, the τd is reported at 28.8 + 3.8 °C; and in environments of 4.3 + 4.9 °C, the τd was reported at 19.1 + 1.0 °C.

The viscosity evaluations of the F1 fish at the ages of 9, 24, and 36 months showed the same tendency of a drastic decrease in the τd above 30 °C, being lower than the τd in the collagen PSC obtained from 36-month-old fish. Due to the effect of high temperatures, collagens change their triple helix structure and fragment into molecules of lower molecular weight [11]. Investigations of τd in tilapia according to age have not previously been evaluated. In order to have a collagen with thermal stability, for preparations of natural products, whether for cosmetic or pharmaceutical use, it is important to consider the age of the fish, the climatic conditions, habitat, and management during rearing.

The concentration of collagen ASC hydroxyproline in samples diluted in ultrapure water showed no differences between collagens from different lines and different ages, which means that the tilapia collagen ASC obtained does not change with age in the hydroxyproline concentration, which supports the τd of collagen ASC in different lines and ages in this research. According to reports, the concentration of proline and hydroxyproline are correlated with the thermal stability of the collagen triple helix [11]. The concentration of these amino acids will depend on the culture conditions, mainly on the water temperature rather than on the genetic lineage [13].

Hydroxyproline was quantified in collagen ASC samples in dilutions with molecular grade water, even when uniform agitation of the collagen samples was carried out, partial dilutions were visible, so the hydroxyproline concentrations are relative and referential values. When making comparisons between lines and ages, no significant differences were observed, with a tendency to decrease Hyp in 36-month-old fish in the three lines. Hyp quantification in PSC collagen was not performed due to prior information on the differential degradation of collagen in the three strains obtained by SDS-PAGE and fractional viscosity.

The structural integrity of the collagen ASC extracted among distinct lineages (red, gray, and F1 progeny) at 9 months was evidenced by the UV absorption spectra. The UV absorption spectra recorded below 240 nm were associated with the purity characteristics of collagens; the absorbance between 220 and 240 nm was related to the C=O, -COOH, and CONH2 groups in the collagen polypeptide chains, with 230 nm being an indicator of a collagen purity 99% [32].

From the FTIR spectra found for the gray, red, and F1 progeny lineages, at the ages of 9, 24, and 36 months, the amides A (3320 cm^−1^), B (2940 to 2930 cm^−1^), I (1640–1630 cm^−1^), II (1540 cm^−1^), and III (1240–1238 cm^−1^) were within the profiles reported for tilapia collagen [31,33]. Such spectra confirmed the integrity of the triple helix structure of collagen in the three genetic lines and among fish of different ages. According to Muyonga et al. [15], the changes in the values of the amides are indicative of variations in the structure of collagen, because of denaturation, to the breaks of the hydrogen bridges mainly in the α chain.

Likewise, the X-ray diffraction diagrams showed two peaks in the diffraction angles clearly differentiated in the three genetic lines at all ages studied, which are indicators of the structural stability of collagen. Sun et al. [31] reported the presence of two peaks for tilapia skin collagen, with values similar to the results obtained in this investigation. From the comparisons of the collagen spectra between the ages of each lineage, there were no significant differences or association with the age of the fish.

The collagen structure of red tilapia and F1 would be an advantage over collagen from gray tilapia, as well as the acetic acid method, for the extraction of collagen from fish, due to the preservation of the stability of the collagen structure, and they also do not require neutralization of the reaction as in the enzymatic method, which is important in the production processes of natural products based on collagen.

## 4. Materials and Methods

### 4.1. Biological Material

*Oreochromis niloticus*, red tilapia lineage (males YY), gray, and F1, obtained from crossing red YY with 9-, 15-, 24-, and 36-month-old grey females were from Faculty of Biological Sciences Experimental Genetics Centre, National University of Trujillo, Trujillo, Peru, breeding temperature 23 ± 3 °C. Species and molecular sexing identification were performed according to Arqueros et al. [21].

Individuals were exposed to thermal shock (−20 °C) to cause a non-painful death. After approximately 30 min, skins were removed by L-cut method. They were firstly washed in potable water until residual meat or scales were eliminated, and lastly immersed in sterile distilled water. All procedures were approved by Institutional Committee of Ethics in research—N° 02-2021).

### 4.2. Collagen Extraction

Extraction was performed by treatments with acetic acid according to Li et al. [18] with some modifications. Skins were soaked 24 h into a 0.1 M NaOH solution in 1:20 (*w*/*v*) proportion. Samples were washed with distilled water until reaching neutral pH and placed in 10% butyl alcohol at 1:20 (*w*/*v*) sample/solution proportion for 24 h. Then, they were washed 10 times with distilled water before proceeding to 0.5 M acetic acid extraction for 48 h. Finally, they were filtered under vacuum using a rapid filtration mesh. The whole procedure was carried out at 4 °C. Then, collagen was extracted by the pepsin enzymatic treatment, obtaining PSC collagen. After skin pretreatment phase, the 0.5 M acetic acid—0.1% pepsin collagen extraction was performed. After 48 h of treatment at 4 °C, samples were vacuum filtered, then they were frozen at −20 °C and lyophilized.

### 4.3. Sodium Dodecyl Sulfate–Polyacrylamide Gel Electrophoresis (SDS-PAGE)

SDS-PAGE was performed according to the method of Laemmli (1970) [34] with a slight modification. Collagen samples (ASC and PSC) lyophilized at 0.5% with 0.1 M acetic acid were mixed with a sample plug (0.5 M Tris-HCl pH 6.8, glycerol, 10% SDS, 1% bromophenol blue, 2-mercaptoethanol, and distilled water) in a 1:1 ratio, heated to 95 °C for 60 s and allowed to cool to room temperature, the standard protein (collagen type I calf skin) was diluted 30 mg in 1 mL of distilled water, then 20 uL of the samples was loaded together with the polyacrylamide gel samples, 7.5% polyacrylamide gel (1.5 M Tris-HCl pH 8.8, 10% SDS, 30% acrylamide/bisacrylamide, 10% APS, TEMED, and distilled water) and 4% concentrating gel (0.5 M Tris-HCl pH 6.8, SDS 10%, acrylamide/bisacrylamide 30%, APS 10%, TEMED, and distilled water). For the running of the bands, an electrophoresis buffer was used (tris base, glycine, SDS, and distilled water), a voltage of 65 V for 6 h, after which time the gels were removed from the chamber (Mini-Protean tetra cell, Bio-Rad) to be stained with brilliant blue of Chromasie R-2550 at 1% (*w*/*v*), acetic acid, and methanol for 5 h, and then to be decolorized with a solution of 96% ethanol and 99.8% acetic acid for 6 h under gentle agitation. Molecular weight determination was performed on the Chemi-doc gel documentator.

### 4.4. Performance

Skin and lyophilized samples were weighted according to the procedure of Bi et al. [35]. Performance estimation was calculated by the following equation:

Relative collagen performance in HB (humid basis) (%) = ((Dry colagen weight)/(Wet fresh skins weight)) × 100

### 4.5. Denaturation Temperature Determination (Based on Viscosity)

The denaturation temperature (τd) was determined considering the average temperature (fractional viscosity) where viscosity changes reached 0.5 in the range of 5 to 40 °C [36]. Fractional viscosity (F (T)) was calculated using the following equation [37]:

Fractional viscosity = (measured viscosity − minimum viscosity/(maximum viscosity − minimum viscosity

### 4.6. Hydroxyproline Quantification

Hydroxyproline quantification was determined by colorimetry using a hydroxyproline assay kit (MAK008, Sigma-Aldrich, St. Louis, MO, USA). It was determined by the reaction of oxidized hydroxyproline with 4-(dimethylamino)benzaldehyde (SMAB). The lyophilized collagens were dissolved in molecular grade water (0.5 mg/mL). The procedure indicated in the insert of the kit was followed. The reading was carried out in a plate reader (iMark^TM^, Microplate Absorbance Reader, Microplate Manager software Bio-Rad, Osaka, Japan) at 560 nm, and the concentration of hydroxyproline in collagen samples was determined using an analytical curve [38].

### 4.7. Ultraviolet (UV) Scanning

Collagens were dissolved in 0.1 M acetic acid. Samples were transferred to quartz cell and UV spectrum profile was determined at 200–400 nm at 24 °C in a Nanodrop C, Thermo Fisher Scientific. An aliquot of 0.1 M acetic acid was used as blank.

### 4.8. Fourier Transform Infrared Spectroscopy (FTIR) Analysis

In order to evaluate the structural characteristics, a Fourier transform infrared spectroscopy analysis was performed using a Thermo Scientific Nicolet iS50. The attenuated total reflectance technique (ATR) was used in a 4000–600 cm^−1^ range with a 4 cm^−1^ resolution [39].

### 4.9. X-ray Diffraction

In order to determinate the type of collagen, their structure was analyzed by X-ray diffraction using a RIGAKU, Miniflex 600 (Japan) powder diffractometer, with a Cu Kα radiation source (λ = 0.154 nm) at a scanning rate of 2°/min in the 2θ range from 5 to 60°, with a size step of 0.02°. The diffractometer operated at 40 kV and 15 mA. 

### 4.10. Statistical Analysis

Data were processed using Origin Professional software. Statistical comparisons were performed with the variance and Tukey analysis of the quantitative data previously adjusted to the normal distribution. Significance level was α = 0.05.

## 5. Conclusions

Total by-products were generated after the filleting of the F1 generation of tilapia at different ages based on total body weight, and the results were obtained in the range of 63.41 to 67.86%, and the percentage of skin in the range of 3.01 to 3, being 55%; hence, the importance of research to give added value to the use of by-products. In relation to the profile of ASC collagen bands, similarity was observed between the red, gray, and F1 lineages and between the different ages. From the observations in SDS-PAGE and the τd in PSC collagen from gray tilapia skin, they show higher degradation and lower τd than those of red tilapia collagen and F1. Although the age of the fish in tilapia farming does not affect the concentration of hydroxyproline, it is important to standardize the age and management conditions in tilapia farming to ensure a stable product. As in the previous evaluations, the ages and lineages did not show significant differences by FTIR and X-ray diffraction, demonstrating, in turn, the acceptable degree of purity for tilapia collagen determined by the visible UV spectrum. Likewise, by means of FTIR and X-ray diffraction, the similarity of tilapia collagen values with the reference (type I bovine collagen) was demonstrated, confirming the purity of the collagen obtained in this research without the need for an additional procedure (purification of collagen), which is very useful when it is necessary to extract large amounts of collagen from fish.

## Figures and Tables

**Table 1:** Weight of fillet and subproducts of F1 XY.

Weight of Fillet and Subproducts	Age (Months)		
	9	15	36
	% w		
Fillet	33.04	29.13	32.84
Subproducts skin weight	3.55	3.01	3.25
Subproducts total weight	63.41	67.86	63.91

**Table 2:** Collagen yield of tilapia skin acid-soluble collagen (ASC) and pepsin-soluble collagen (PSC) of the red, gray, and F1 lineages, 9-months old (*p* > 0.05).

Collagen Extraction Method	Skin Weight (g)	Lyophilized Collagen Weight (g)	Yield (%)
ASC Red	8.93 ± 0.61	1.70 ± 0.16	19.04 ± 0.59
ASC Gray	6.93 ± 1.37	1.39 ± 0.12	20.06 ± 2.20
ASC F1	8.53 ± 0.42	1.73 ± 0.16	20.28 ± 1.86
PSC Red	8.53 ± 0.42	1.80 ± 0.12	21.10 ± 1.59
PSC Gray	6.93 ± 1.37	1.47 ± 0.29	21.21 ± 1.14
PSC F1	8.53 ± 0.42	1.80 ± 0.12	21.10 ± 1.59

**Table 3:** Distribution of infrared spectroscopy of acid-soluble collagen extracted from the skin of tilapia (*Oreochromis niloticus*). CCS (collagen from calf skin—Type I); R-9, G-9, F1-9 (red, gray, and F1 of 9 months), R-15, G-15, F1-15 (red, gray, and F1 of 15 months); R-24, G-24, F1-24 (red, gray, and F1 of 24 months); R-36, G-36, F1-36 (red, gray, and F1of 36 months).

Region	CCS	R-9	G-9	F1-9	R-15	G-15	F1-15	R-24	G-24	F1-24	R-36	G-36	F1-36
Amide A	3304	3290	3290	3290	3295	3295	3294	3290	3290	3290	3292	3290	3290
Amide B	2925	2940	2940	2940	2933	2939	2929	2945	2940	2930	2931	2931	2930
Amide I	1631	1640	1640	1630	1632	1633	1631	1630	1640	1630	1632	1632	1631
Amide II	1547	1540	1540	1540	1545	1545	1544	1540	1540	1540	1542	1540	1540
Amide III	1235	1240	1240	1240	1238	1238	1238	1240	1240	1240	1240	1239	1239

**Table 4:** X-ray diffraction pattern estimates of tilapia skin collagen samples at 9, 15, 24, and 36 months.

Strain Collagen	Months	Peak 1 (°)	Peak 2 (°)
STD		7.15	20.64
Gray	9	7.25	21.80
	15	7.47	21.17
	23	7.48	21.86
	36	7.54	21.60
Red	9	7.47	21.00
	15	7.47	20.96
	23	7.70	21.58
	36	7.40	21.15
F1 (H)	9	7.33	20.82
	15	7.78	21.28
	23	7.46	21.44
	36	7.44	21.51

## Data Availability

The data can be requested via email (zprieto@unitruedu.pe) when the interested parties request it.

## Sample Availability

Samples of the compounds are not available from the authors.

## References

[1] León-López A., Morales-Peñaloza A., Martínez-Juárez V.M., Vargas-Torres A., Zeugolis D.I., Aguirre-Álvarez G. (2019). Hydrolyzed Collagen—Sources and Applications. Molecules.

[2] Lupi O. (2002). Prions in dermatology. J. Am. Acad. Dermatol..

[3] Fang T.H., Lien C.Y. (2020). Mini review of trace metal contamination status in East China Sea sediment. Mar. Pollut. Bull..

[4] Esiukova E., Zobkov M., Chubarenko I. (2019). Data on microplastic contamination of the Baltic Sea bottom sediment samples in 2015–2016. Data Brief.

[5] Barboza L.G.A., Lopes C., Oliveira P., Bessa F., Otero V., Henriques B., Raimundo J., Caetano M., Vale C., Guilhermino L. (2019). Microplastics in wild fish from North East Atlantic Ocean and its potential for causing neurotoxic effects, lipid oxidative damage, and human health risks associated with ingestion exposure. Sci. Total Environ..

[6] Kakimoto S., Yoshimitsu M., Akutsu K., Kiyota K., Fujiwara T., Watanabe T., Kajimura K., Yamano T. (2019). Concentrations of total mercury and methylmercury in red snow crabs (*Chionoecetes japonicus*) caught off the coast of Japan. Mar. Pollut. Bull..

[7] FAO-Food and Agriculture Organization of the United Nations the State of World Fisheries and Aquaculture. http://www.fao.org/3/i9540en/i9540en.pdf.

[8] Hemker A.K., Nguyen L.T., Karwe M., Salvi D. (2019). Effects of pressure-assisted enzymatic hydrolysis on functional and bioactive properties of tilapia (*Oreochromis niloticus*) by-product protein hydrolysates. LWT.

[9] Ramshaw J.A.M., Shah N.K., Brodskyb B. (1998). Gly-X-Y Tripeptide Frequencies in Collagen: A Context for Host–Guest Triple-Helical Peptides. J. Struct. Biol..

[10] Berisio R., Granata V., Vitagliano L., Zagari A. (2004). Imino Acids and Collagen Triple Helix Stability: Characterization of Collagen-like Polypeptides Containing Hyp-Hyp-Gly Sequence Repeats. J. Am. Chem. Soc..

[11] Xu S., Gu M., Wu K., Li G. (2019). Unraveling the Role of Hydroxyproline in Maintaining the Thermal Stability of the Collagen Triple Helix Structure Using Simulation. J. Phys. Chem. B.

[12] Ghanaeian A., Soheilifard R. (2018). Mechanical elasticity of proline-rich and hydroxyproline-rich collagen-like triple-helices studied using steered molecular dynamics. J. Mech. Behav. Biomed. Mater..

[13] Akita M., Nishikawa Y., Shigenobu Y., Ambe D., Morita T., Morioka K., Adachi K. (2020). Correlation of proline, hydroxyproline and serine content, denaturation temperature and circular dichroism analysis of type I collagen with the physiological temperature of marine teleosts. Food Chem..

[14] Thompson H.C., Contin R.F. (1980). Changes in the concentration of free and collagen-bound hydroxyproline in muscle tissue of the bluehead wrasse (*Thalassoma bifasciatum*) as a function of age. Comp. Biochem. Physiol. Part A Physiol..

[15] Muyonga J.H., Cole C.G.B., Duodu K.G. (2004). Characterisation of acid soluble collagen from skins of young and adult Nile perch (*Lates niloticus*). Food Chem..

[16] Prieto L.Z.A., Salirrosas F.R.D., Arqueros A.M., Sanchez T.L.C., Gastañadui R.D., Fernádez R.R. (2018). Obtención de Machos YY de Oreochromis Niloticus, Tilapia.

[17] Gómez-Marquez J.L., Peña-Mendoza B., Salgado-Ugarte I.H., Arredondo-Figueroa J.L. (2008). Age and growth of the tilapia, Oreochromis niloticus (*Perciformes: Cichlidae*) from a tropical shallow lake in Mexico. Rev. Biol. Trop..

[18] Li J., Wang M.C., Qiao Y., Tian Y.Y., Liu J.H., Qin S., Wu W.H. (2018). Extraction and characterization of type I collagen from skin of tilapia (*Oreochromis niloticus*) and its potential application in biomedical scaffold material for tissue engineering. Process. Biochem..

[19] FAO-Food and Agriculture Organization of the United Nations Cultured Aquatic Species Information Programme. https://www.fao.org/fishery/collection/cultured-species/en.

[20] Romana-Eguia M.R.R., Ikeda M., Basiao Z.U., Taniguchi N. (2004). Genetic diversity in farmed Asian Nile and red hybrid tilapia stocks evaluated from microsatellite and mitochondrial DNA analysis. Aquaculture.

[21] Arqueros M., Tuesta L.S., Prieto Z. (2017). Diferenciación genética de tilapia roja y gris (*Oreochromis niloticus*) mediante microsa- télites y marcadores SCAR como indicadores del sexo genético. Rev. Peru. Biol..

[22] Parichy D.M. (2021). Evolution of pigment cells and patterns: Recent insights from teleost fishes. Curr. Opin. Genet. Dev..

[23] Wang L.-M., Luo M.-K., Yin H.-R., Zhu W.-B., Fu J.-J., Dong Z.-J. (2020). Effects of background adaptation on the skin color of Malaysian red tilapia. Aquaculture.

[24] Alfaro A.D.T., Fonseca G.G., Balbinot E., Machado A., Prentice C. (2013). Physical and chemical properties of wami tilapia skin gelatin. Food Sci. Technol..

[25] Le T.M.T., Nguyen V.M., Tran T.T., Takahashi K., Osako K. (2020). Comparison of acid-soluble collagen characteristic from three important freshwater fish skins in Mekong Delta Region, Vietnam. J. Food Biochem..

[26] Ogawa M., Moody M.W., Portier R.J., Bell J., Schexnayder A.M.A., Losso J.N. (2003). Biochemical Properties of Black Drum and Sheepshead Seabream Skin Collagen. J. Agric. Food Chem..

[27] Zhang M., Liu W., Li G. (2009). Isolation and characterisation of collagens from the skin of largefin longbarbel catfish (Mystus macropterus). Food Chem..

[28] Chen S., Tang L., Su W., Weng W., Osako K., Tanaka M. (2015). Separation and characterization of alpha-chain subunits from tilapia (*Tilapia zillii*) skin gelatin using ultrafiltration. Food Chem..

[29] Kimura S., Ohno Y., Miyauchi Y., Uchida N. (1987). Fish skin type I collagen: Wide distribution of an α3 subunit in teleosts. Comp. Biochem. Physiol. Part B Biochem..

[30] Gistelinck C., Gioia R., Gagliardi A., Tonelli F., Marchese L., Bianchi L., Landi C., Bini L., Huysseune A., Witten P.E. (2016). Zebrafish Collagen Type I: Molecular and Biochemical Characterization of the Major Structural Protein in Bone and Skin. Sci. Rep..

[31] Sun L., Hou H., Li B., Zhang Y. (2017). Characterization of acid- and pepsin-soluble collagen extracted from the skin of *Nile tilapia* (*Oreochromis niloticus*). Int. J. Biol. Macromol..

[32] Pal G.K., Suresh P. (2017). Comparative assessment of physico-chemical characteristics and fibril formation capacity of thermostable carp scales collagen. Mater. Sci. Eng. C.

[33] Chen J., Li L., Yi R., Xu N., Gao R., Hong B. (2016). Extraction and characterization of acid-soluble collagen from scales and skin of tilapia (*Oreochromis niloticus*). LWT.

[34] Laemmli U.K. (1970). Cleavage of Structural Proteins during the Assembly of the Head of Bacteriophage T4. Nature.

[35] Bi C., Li X., Xin Q., Han W., Shi C., Guo R., Shi W., Qiao R., Wang X., Zhong J. (2019). Effect of extraction methods on the preparation of electrospun/electrosprayed microstructures of tilapia skin collagen. J. Biosci. Bioeng..

[36] Nagai T. (2000). Isolation of collagen from fish waste material—skin, bone and fins. Food Chem..

[37] Bhagwat P., Dandge P. (2016). Isolation, characterization and valorizable applications of fish scale collagen in food and agriculture industries. Biocatal. Agric. Biotechnol..

[38] Huang C.-Y., Kuo J.-M., Wu S.-J., Tsai H.-T. (2016). Isolation and characterization of fish scale collagen from tilapia (*Oreochromis* sp.) by a novel extrusion–hydro-extraction process. Food Chem..

[39] Giraldo-Rios D.E., Rios L.A., Montoya J.E.Z. (2020). Kinetic modeling of the alkaline deproteinization of Nile-tilapia skin for the production of collagen. Heliyon.

